# L-Citrullinato-Bipyridine and L-Citrullinato-Phenanthroline Mixed Copper Complexes: Synthesis, Characterization and Potential Anticancer Activity

**DOI:** 10.3390/pharmaceutics16060747

**Published:** 2024-05-31

**Authors:** Diego Ramírez-Contreras, Sergio Vázquez-Rodríguez, Amalia García-García, Lisset Noriega, Angel Mendoza, Brenda L. Sánchez-Gaytán, Francisco J. Meléndez, María Eugenia Castro, Maura Cárdenas-García, Enrique González-Vergara

**Affiliations:** 1 Centro de Química, Instituto de Ciencias, Benemérita Universidad Autónoma de Puebla, 18 sur y Av. San Claudio, Col. San Manuel, Puebla 72570, Mexico; diego.ramirezcontreras@alumno.buap.mx (D.R.-C.); sergio.vazquezrodriguez@alumno.buap.mx (S.V.-R.); amaliagarcia@ugr.es (A.G.-G.); angel.mendoza@correo.buap.mx (A.M.); brenda.sanchez@correo.buap.mx (B.L.S.-G.); mareug.castro@correo.buap.mx (M.E.C.); 2 Departamento de Química Inorgánica, Facultad de Ciencias, Universidad de Granada, Av. Fuente Nueva s/n, 18003 Granada, Spain; 3 Departamento de Física Aplicada, Centro de Investigación y de Estudios Avanzados, Unidad Mérida, km 6 Antigua Carretera a Progreso, Apdo. Postal 73, Cordemex, Mérida 97310, Mexico; lis.noriega.santos@gmail.com; 4 Facultad de Ciencias Químicas, Benemérita Universidad Autónoma de Puebla, 18 sur y Av. San Claudio, Col. San Manuel, Puebla 72570, Mexico; francisco.melendez@correo.buap.mx; 5 Laboratorio de Fisiología Celular, Facultad de Medicina, Benemérita Universidad Autónoma de Puebla, 13 sur 2702, Puebla 72410, Mexico

**Keywords:** citrulline, anticancer, mixed-copper complexes, 1,10-phenanthroline, 2,2-bipyridine

## Abstract

Citrulline (C_6_H_13_N_3_O_3_) is an amino acid found in the body as a zwitterion. This means its carboxylic and amine groups can act as Lewis donors to chelate metal cations. In addition, citrulline possesses a terminal ureido group on its aliphatic chain, which also appears to coordinate. Here, two new mixed complexes of citrulline were made with 1,10-phenanthroline and 2,2′-bipyridine. These compounds, once dissolved in water, gave aquo-complexes that were subject to DFT studies and in vitro toxicity studies on cancer cell lines (HeLa, MDA-MB-231, HCT 15, and MCF7) showed promising results. Docking studies with DNA were also conducted, indicating potential anticancer properties.

## 1. Introduction

**Figure 1:**
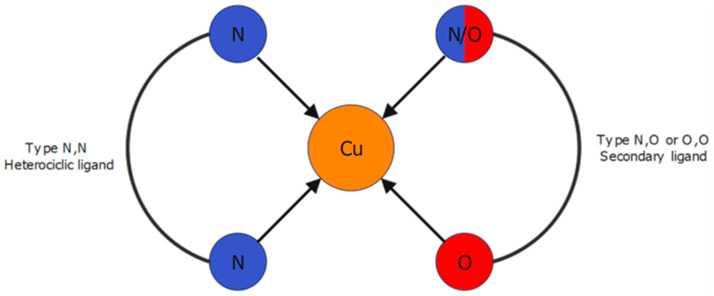
General representation of Casiopeina structure, based on the Ruiz-Azuara work.

Since the discovery of the antineoplastic properties of Cisplatin by Rosenberg’s accidental experiment in the 1960s [1,2,3], coordination chemistry has expanded its areas in medicine beyond the simple study of metal salts to which it seemed to be confined. Although Cisplatin is the metallodrug of choice for a broad spectrum of malignancies, its use has many drawbacks [4,5,6,7,8]. Unfortunately, only a few molecules have been approved for the many coordination complexes expected to replace cisplatin [9,10,11]. Among the few successful cases, Casiopeinas (Figure 1) have stood out for their excellent antineoplastic properties and high DNA binding affinity [12,13,14]. Several Casiopeina-like compounds have been prepared with different ligands to find suitable candidates for clinical trials, always seeking to meet the biocompatibility requirement. On the other hand, amino acids are the most studied ligands for this purpose due to their biological involvement in metabolic pathways and well-known chelating ability [15].

Intrinsic amino acids in metabolic pathways attract attention as proposals for new complexes. One corresponds to citrulline, a non-essential, non-protein amino acid related to the urea cycle and arginine synthesis in the body [16]. Despite the widespread disinterest in this compound beyond arginine metabolism, in recent years, this amino acid has been identified as an essential regulatory factor in the blood pressure process [17]. It is directly related to NO regulation in the blood via the conversion of arginine [18]. Furthermore, the role of this amino acid in some autoimmune disorders, such as rheumatoid arthritis and lupus, is directly associated with a natural post-translational process of proteins called citrullination, where arginine residues in proteins are converted into citrulline [19]. In patients with these disorders, antibodies attack these post-translational products [20]. Therefore, the study of this amino acid has gained more weight in several recent investigations. Also, the toxicity of L-citrulline on HeLa cells was demonstrated in a manner that depended on the dosage. According to the results obtained from Annexin and Caspase experiments, it can be inferred that L-citrulline had a pro-apoptotic impact on HeLa cells, but only when exposed for a brief period of time. L-citrulline also exhibited a migratory inhibitory effect. The results of this study suggest that L-citrulline should be further examined for its potential anticancer effects in laboratory and animal models, as well as as a potential treatment option for cancer [21].

Discovered by Wada in 1914, its identification was made possible thanks to the synthesis of one of its complexes, copper (II) bis-citrullinato [Cu(Citr)_2_] [22,23]. The citrulline complex has been well-known for a long time but has attracted little interest since most of the research on this compound was described as “difficult to crystallize” [24]. This explains the widespread disinterest in citrulline complexes; e.g., in the Cambridge Crystallographic Database, there are only three reported crystal structures for citrulline compounds (1048343, 2172421, and 2287583). The structure of the palladium complex, described by Mascaliovas [25], was determined using powder X-ray diffraction, whereas most investigations have been conducted in solution [26,27,28,29,30,31]. Our research group has provided the other two structures.

Over three decades have passed since the inception of Casiopeinas, and substantial evidence has accumulated concerning the mechanisms by which these compounds operate. These molecules can potentially be used in cancer therapy because they can damage mitochondria and increase reactive oxygen species, which damage DNA [12,13,14,32,33]. The polypharmacological characteristics of these molecules guarantee their continued relevance as viable alternatives for treating ailments such as cancer [34]. Based on the Lena Ruiz research group’s preclinical results and expertise, Casiopeina (CasIIIia) has been submitted to the Mexican regulatory agency (COFEPRIS). This signifies the commencement of the initial Phase I clinical trial in Mexico involving a copper-based anticancer compound [35]. Recently, considerable scientific attention has been directed towards these molecules because of their biological activities and chemical properties [36,37]. 

As mentioned above, despite the intrinsic difficulties of crystallization of citrulline, previous works from our laboratory have reported the structures from single crystal XRD of copper(II)bis-citrullinato and aquabipyridinecitrullinatonitratocopper(II) monohydrate [24,38], The landmarks in these findings lie in the ability of citrulline to form polymeric structures and the ability of copper to isomerize citrulline from L to D stereoisomer. This information, together with in-silico research, shows that the amino acid complexes promise to exhibit biological activity. The synthesis in methanol resulted in interesting polymeric structures that, upon recrystallization, generated monomeric species that were studied by the DFT method. Here, we report two new catena complexes [Cu(L-Citr)(bipy)(NO_3_)]_n_ (Complex **1**) and [Cu(L-Citr)(phen)(NO_3_)]_n_ (Complex **2**), that were fully characterized through spectroscopic and computational techniques and their biological capabilities were analyzed through in vitro and cytotoxic assays. Importantly, docking studies with relevant proteins and In vitro toxicity studies on cancer cell lines (HeLa, MDA-MB-231, HCT 15, and MCF7) showed potential anticancer properties.

## 2. Materials and Methods

### 2.1. Materials

All the reagents (ACS purity), media, solvents, and biological supplies (DNA from calf thymus) were purchased from Sigma-Aldrich (Merck KGaA, Darmstadt, Germany). The pUC19 plasmid was acquired by OXYGENE (Oxford Genetics Ltd., Oxford, UK). The plasmid was expressed in the *E. Coli* HB101 strain (Bio-Rad Laboratories, Inc., Hercules, CA, USA) and purified via alkaline lysis with the GenElute^®^ plasmid miniprep kit (Merck KGaA, Darmstadt, Germany). Cancer cell lines were provided by Laboratorio de Fisiologia Celular from the Faculty of Medicine of the Benemérita Universidad Autónoma de Puebla (BUAP). The cell lines belonged to the ATCC (Manassas, VA, USA) cell culture collection and were maintained according to the supplier’s recommendations [39]. 

### 2.2. Equipment

The UV-Vis studies in solution were conducted using a Cary 50 spectrophotometer (Agilent Technologies, Santa Clara, CA, USA), utilizing a quartz cuvette with a width of 10 mm for measuring concentrations. A Nicolet 6700 FTIR spectrophotometer (Thermo Fisher Scientific, Waltham, MA, USA), equipped with the iTR attachment featuring a diamond tip, was used to capture the infrared spectra in the range of 4000 to 650 cm^−1^. X-ray diffraction tests were conducted on high-quality crystals. The data was gathered using an Oxford Diffraction Gemini-Atlas diffractometer (Oxford Instruments, Oxfordshire, UK) that had a charge-coupled device area detector and used graphite monochromated Mo-Kα radiation (λ = 0.71073 Å). The electrophoresis experiments were conducted using a Bio-Rad Mini-Sub Cell GT Cell electrophoresis (Bio-Rad Laboratories, Inc., Hercules, CA, USA) chamber equipped with a Daigger Biotech 300 power supply (Daigger, Vernon Hills, IL, USA). Fluorometric assays were performed on a Varian Cary Eclipse fluorescence spectrometer (Agilent Technologies, Santa Clara, CA, USA). 

### 2.3. Software Crystallography

Data collection and absorption correction were performed using the CrysAlis PRO and CrysAlis RED software packages [40]. The structure was resolved using direct approaches with the ShelXT software and subsequently refined through full-matrix least-squares on F2 with SHELXL-2019 [41]. Refinement was conducted on the positional and anisotropic atomic displacement parameters of all non-hydrogen atoms. The hydrogen atoms were identified in various Fourier maps and accounted for as fixed components attached to their parent atoms. The isotropic thermal factors for the hydrogen atoms were selected to be 1.2 times that of their parent atoms. The Olex2 program [42] served as the graphical interface. The crystallographic data for the structure described in this research have been submitted to the Cambridge Crystallographic Data Center (CCDC 23418117, 2341818).

### 2.4. Synthesis of Complexes

#### 2.4.1. Synthesis of [Cu(L-Citr)(bipy)(NO_3_)]_n_ (Complex **1**) 

The synthesis of Complex **1** was performed in the same manner described in previous work [24]. In 30 mL of a methanolic mixture of citrulline (C_6_H_13_N_3_O_3_) (1 mmol) and copper nitrate monohydrate (Cu(NO_3_)_2_∙3H_2_O) (1 mmol), 1 mL of 2,2′-bipyridine (C_10_H_8_N_2_) in methanol (1 M) was added, the pH was adjusted to 7–8, the resulting solution was filtered and leave to crystallize at room temperature, but in this case the product did not recrystallize in water. After one day, anhydrous blue crystalline needles were isolated, but the product was not recrystallized in water. The anhydrous blue crystalline needles were obtained. FT-IR (ATR, cm^−1^): 3460m, 3329m, 3281m, 3226w, 3083w, 2944w, 1669s, 1582br, 1558br, 1323vs, 1311vs, 773s. UV-Vis (ε_max_, M^−1^ cm^−1^): 300 nm (1.43 × 10^4^), 312 nm (1.35 × 10^4^), 605 nm (60).

#### 2.4.2. Synthesis of [Cu(L-Citr)(phen)(NO_3_)]_n_ (Complex **2**)

The synthesis of Complex **2** was performed in the same way that Complex 1, using 1,10-phenanthroline (C_12_H_8_N_2_) as a tertiary ligand instead of bipyridine. After one day, deep blue crystal clusters were isolated. FT-IR (ATR, cm^−1^): 3393m, 3319m, 3257m, 3218w, 2925w, 1644w, 1597br, 1582br, 1554s, 1385s, 1315s, 852s, 724s. UV-Vis (ε_max_, M^−1^ cm^−1^): 272 nm (3.20 × 10^4^), 294 nm (1.02 × 10^4^), 615 nm (52).

### 2.5. Quantum Mechanical Calculations

Calculations based on the density functional theory, DFT [43], were carried out to analyze the non-polymeric complexes’ molecular structure, electronic properties, and non-covalent interactions. The PBEPBE functional [44] was used with the LANL2DZ basis set [45]. The solvent effect, using water as a solvent, was implicitly included in the universal solvation model (SMD) [46]. The vibrational frequencies were calculated to ensure minimum structures on the potential energy surface (PES). Two structures were modeled to test their behavior in solution: Complexes **1′** and **2′**. In both, the H_2_O molecule was placed at the top of a distorted square pyramid. The molecular electrostatic potential (MEP) maps were analyzed for the two systems. The calculations were carried out with the Gaussian 16 program [47]. The results were visualized with the Gaussian View 6.0.16 program [48]. The main non-covalent interactions were characterized using the atoms in molecules (AIM) approach with the AIMAll software 19.10.12 [49].

### 2.6. Docking Studies with AutoDock 4.2

A molecular docking study was carried out using AutoDock 4.2 software to explore the potential interactions of complexes with two different DNA targets [50]. The crystal structures of the targets were obtained from the RSCB Protein Data Bank (PDB) [51]. The PDB IDs 1BNA and 151D [52,53] were used to evaluate the complexes’ binding interactions with DNA using AutoDock Tools. The macromolecule and its ligands were prepared by removing the water molecules, adding polar hydrogens, and setting the Gasteiger charges. The receptor grid box was set according to the targets studied, as follows: for 1BNA, the box was centered at x = 14.78 Å, y = 20.976 Å, and z = 8.807 Å with a box size of 70 × 70 × 120 Å3, the box used for 151D was centered at x = 14.385 Å, y = 13.535 Å, z = 13.098 Å with a box size of 70 × 70 × 70 Å3. Docking studies were conducted using a population size of 150 individuals, a maximum energy evaluation of 2,500,000, and a maximum generation of 27,000 to result in 50 docking poses. The parameters for the copper(II) compounds during the docking procedure followed the protocol outlined in our previous studies [54,55], where the force field used was a semiempirical free energy scoring function that considers the contribution of the hydrogen bonds and the electrostatic interactions. The corresponding interaction figures were prepared using Biovia Discovery Studio Visualizer v21.1.0 [56] and VMD v1.9.4 software [57]. 

### 2.7. In-Vitro Assays 

#### 2.7.1. Affinity for CT-DNA

The ability of complexes to bind DNA was studied via spectrophotometry titrations with pure CT-DNA solutions (A_280_/A_260_ ≥ 1.8) (ε_260_ = 6600 cm^−1^ M^−1^ [58]). Using 1 mL of 10 μM solutions of every complex in TBS buffer (1× pH = 7.4) in a 1 cm path quartz cuvette, a solution of 10 μM CT-DNA in the same buffer was added to the complex solutions in different micro-volumes for every point in the titration curve, and the changes produced in the UV-Vis spectra of the complex were recorded. The changes produced in molar absorptivity constants (ε_max_) in the spectra of the complex were mathematically modeled using the Wolfe-Schimer model of the Benesi-Hildebrand equation [59] to estimate the affinity constant (K_b_) (Equation (1)).

(1) $$\frac{\left[DNA\right]}{∆\varepsilon_{\left(a-f\right)}}=\frac{\left[DNA\right]}{∆\varepsilon_{\left(b-f\right)}}+\frac{1}{K_{b}∆\varepsilon_{\left(a-f\right)}}$$

where [*DNA*] is the CT-DNA concentration, and ∆ε_(a−f)_ and ∆ε_(b−f)_ are the differences in the absorptivity coefficients between the apparent and fully bound states of the complex concerning the free state, respectively. The parameters were calculated using a linear regression model in a plot [*DNA*] vs. [*DNA*]/∆ε_(a−f)_ by clearing them from the slope and intercept.

#### 2.7.2. Fluorometric Assays

Fluorometric titrations of the EB-*_ds_*DNA system with the complexes were used to study how well the complexes could displace the strong DNA intercalator ethidium bromide (EB) out of the EB-*_ds_*DNA system. This assay is an excellent test to help elucidate the binding mode between the DNA and the complexes [60]. The EB-*_ds_*DNA system was prepared by mixing CT-DNA (A_280_/A_260_ ≥ 1.8) and EB in TBS buffer (1x, pH = 7.4) to achieve a concentration of 10 μM and 0.37 μM, respectively. Then, 1 mL of the mix, equilibrated at room temperature for 5 min in the dark, was titrated with a solution (10 μM) of the tested complex (always using the same buffer). The changes produced in the fluorescent spectra (550–700 nm, Ex: 525 nm) were recorded at every point in the titration curve. If a decrease in the intensity of the system spectrum is observed, intercalation may be inferred as the mode of DNA binding due to the displacement of the ethidium bromide, which breaks the enhanced intensity provided by this intercalator when it binds DNA in this mode [61]. The differences in intensity between the EB-*_ds_*DNA system free and titrated with the complex were mathematically modeled using the Stern-Volmer model for quenching studies [62], applied for this case, which takes the following form (Equation (2)).

(2) $$\frac{I_{0}}{I}=1+k_{q}\tau_{0}\left[CuLL\right]=1+K_{sv}[CuLL]$$

where *I*_0_ and *I* represent the maximal intensity of the system between zero and every point in the titration curve, respectively, the complex concentration [*CuLL*] is at every point in the titration curve; *k_q_* is the quenching constant for the tested complex; *τ*_0_ is the system’s lifetime; and *K_sv_* is the Stern-Volmer constant for the tested complex. This model provides a Stern-Volmer plot when the intensity quotient is plotted against complex concentration, using the linear regression model with an intercept equal to 1 and a slope equal to *K_sv_*.

#### 2.7.3. Interaction with pUC19 Plasmid

The ability of complexes to bind and cleave plasmid DNA was studied in the pUC19 plasmid by electrophoresis. A sample of 300 ng of pUC19 plasmid in 20 μL of nuclease-free water was incubated at 37 °C for 24 h with the metal complex at different concentrations (5–100 μM). The samples were run in a 1% agarose gel stained with ethidium bromide (EB) (10 mg/mL) (5 μL EB/100 mL agarose gel) over TAE buffer (1x pH = 7.4) at 100 V and 300 mA for 1 h. Once electrophoresis was complete, the gel was revealed in a UV transilluminator, monitoring the changes in the plasmid bands produced by complex action. A typical plasmid sample exhibits the supercoiled form (Form I) and the relaxed coil form (Form II). However, if a cleavage in the structure is made by complex action, a third band would be expected to be observed between the two forms, which corresponds to the lineal form of a plasmid (Form III) [63,64].

#### 2.7.4. Cytotoxic Assays

The cytotoxicity of complexes was tested in four cancer cell lines: HeLa (cervix cancer), HCT-15 (colorectal cancer), MDA-MB-231, and MCF-7 (breast cancer). The cells were grown in appropriate media (see Table S1) supplemented with 5% fetal bovine serum (FBS) and 100 U/0.1 mg/mL of penicillin/streptomycin antibiotic mix at 37 °C under a 5% CO_2_ atmosphere in triplicate, each cell line to test was seeded in 96-well plates (3 × 10^3^ cells per well) and incubated under the same conditions described previously. Subsequently, the media was aspirated and replaced with media supplemented with a filtered (0.25 μm) solution of each tested complex, increasing the concentration (1–10 μM) in each well row from the positive and negative control well rows. Finally, the plates were incubated for another 24 h under the same conditions described above, and the percentage of viable cells was measured by the XTT colorimetric assay [65] using a microplate spectrophotometer. The absorbance values (*ABS_i_*) were transformed to the percentage of response (dead cell population) (*R_i_*) with the relationship described in Equation (3):

(3) $$R_{i}=1-\frac{{ABS}_{i}-{ABS}_{nc}}{{ABS}_{pc}-{ABS}_{nc}}$$

The average absorbance values of the negative (*ABS_nc_*) and positive (*ABS_pc_*) controls are those produced by fully viable and fully non-viable populations, respectively. The transformed values give a dose-response curve, which can be numerically fitted with the Hill equation using the Sigma Plot v.12.0 (Systat Software, Inc., San Jose, CA, USA), which allows calculating the IC_50_ parameter.

## 3. Results

### 3.1. Molecular Structures

Complexes **1** and **2** are related compounds containing the amino acid citrulline and nitrogen-based heterocycles (i.e., bipyridine or phenanthroline) as ligands. Both complexes present similar asymmetric units, but variances are shown in the three-dimensional arrangement. 

**Figure 2:**
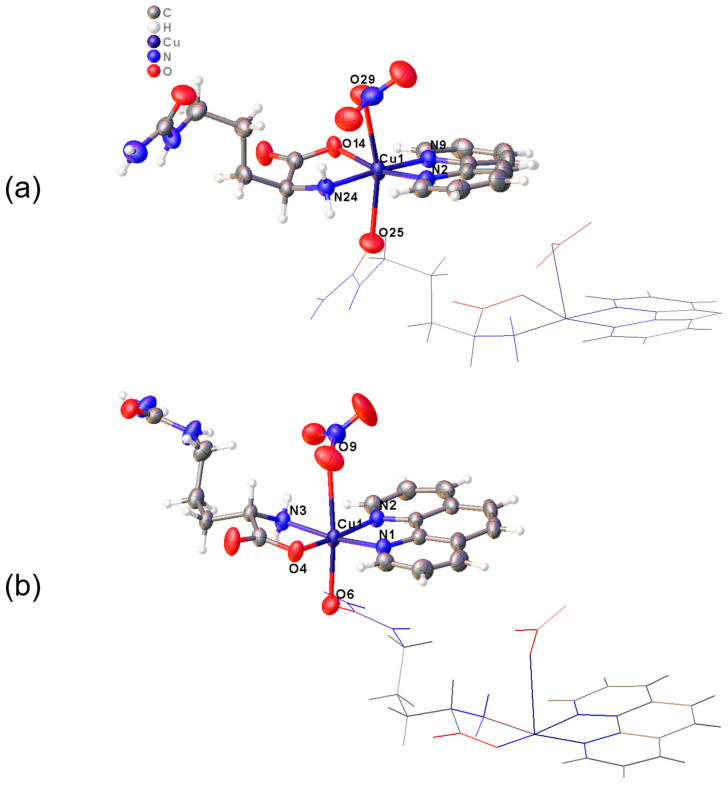
Detail of the coordination spheres of (**a**) Complex **1**, [Cu(L-Citr)(bipy)(NO_3_)]_n_, and (**b**) Complex **2**, [Cu(L-Citr)(phen)(NO_3_)]_n_.

Crystals of Complex **1** comprise polymeric chains parallel to the “*b* crystallographic axis”. Complex **2** comprises chains interlaced and connected by hydrogen bonds (Table 1). The asymmetric units of both compounds contain one Cu(II) ion in a distorted octahedral geometry coordinated to one bipy or phen ligand and one citrulline in the equatorial plane. On the other hand, one nitrate ion and another citrulline molecule belonging to an adjacent molecule occupy the apical positions (Figure 2). This causes the complexes to acquire a polymeric nature. 

The coordination spheres in both complexes undergo deformation due to the splitting of energy of the degenerate d-orbitals caused by the Jahn-Teller effect [66]. In Complex **1**, the equatorial plane’s Cu-N_eq_ and Cu-O_eq_ bonds acquire length values of 1.983(2)–2.001(2) and 1.952(2) Å, respectively. However, because of the Jahn-Teller effect, the Cu-O_ap_ (O28, O25) bond distances in the apical positions are larger, ranging from 2.443(2) to 2.668(3) Å values. Similarly, Complex **2** undergoes the same effect, with Cu-N_eq_ and Cu-O_eq_ bond distances in the range of 1.990(4)–2.044(3) Å and 1.939(3)–1.942(3) Å, respectively, and Cu-O_ap_ (O9, O6) bond lengths of 2.340(3)–2.784(4) Å. All values are nearly identical in both molecules and similar to the hydrated complex [Cu(Citr)(bipy)(H_2_O)(NO_3_)]·(H_2_O), previously reported by us [24].

**Figure 3:**
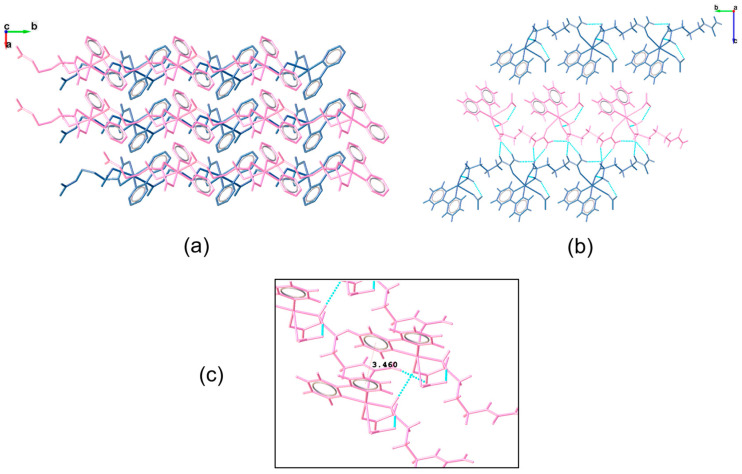
Structure of Complex **1**. (**a**) Chains run parallel to the *b*-axis. Hydrogen atoms have been omitted for clarity; (**b**) as a result of the 2-fold axes, the pair of chains interact by N-H···O hydrogen bonds; (**c**) п-п stacking interactions exist between aromatic rings of equivalent chains.

The polymeric chains formed by these complexes are similar, but their arrangements are entirely different. Complex **1**, due to the 2-fold axis in its structure, forms two parallel chains along the “*b*-axis”, with bipy and citrulline ligands pointing outwards and inwards, respectively (Figure 3). In this configuration, the pair of chains interact through N-H···O hydrogen bonds between the amines and carboxylate groups of citrulline molecules (Table 2). Moreover, chains interact with one another along the “*a* axis” through hydrogen bonds between the carboxylate group of citrulline and the amine group of the α-carbon of adjacent chains, as well as by п-п stacking interactions (3.460 Å length) between bipyridine rings. Along the “*c*-axis”, the pair of chains is isolated.

**Figure 4:**
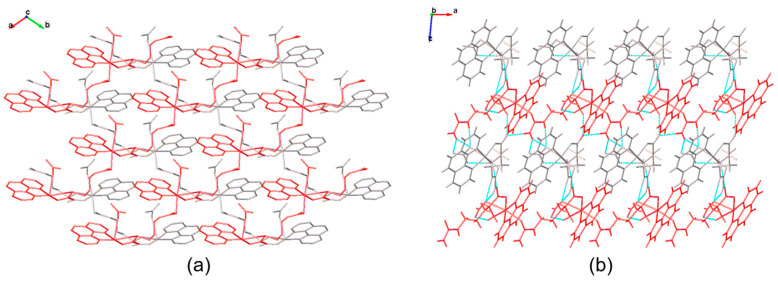
Structure of Complex **2**. (**a**) Chains are intercalated, resulting in a grill-shaped configuration. Hydrogen atoms have been omitted for clarity; (**b**) a hydrogen bond network joins all the chains.

On the other hand, Complex **2** contains two non-equivalent Cu(II) ions, which create two different kinds of polymeric chains, named A and B. Chains A and B run parallel to the “*a* and *b* crystallographic axes”, respectively. In addition, chains of the same type are arranged in a laminated shape, as shown in Figure 4. This configuration results in a grill-shaped structure. Further, an intricate network of N-H···O hydrogen bonds that hold chains A and B together stabilizes the chains (Table 3).

### 3.2. Molecular Structure and Non-Covalent Interactions

**Figure 5:**
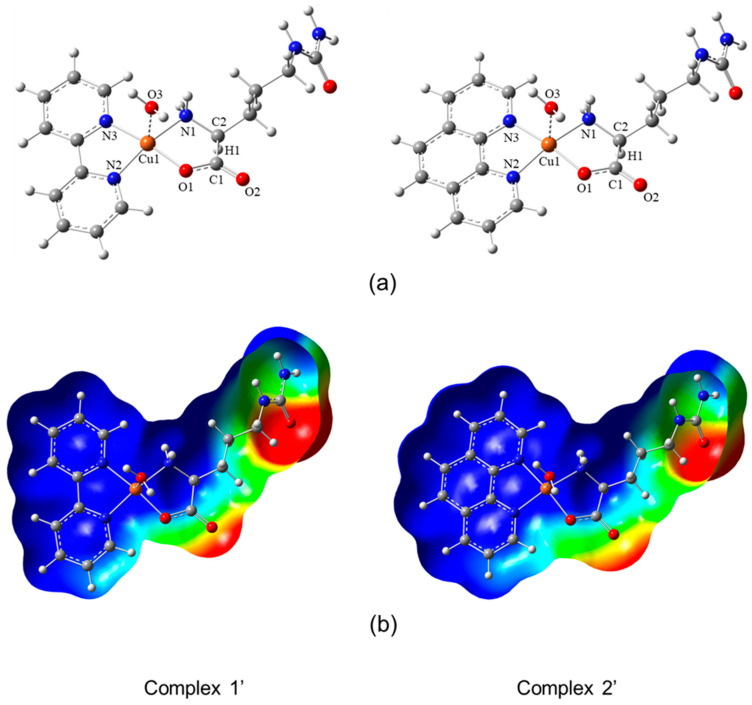
(**a**) Optimized Complex **1′** and Complex **2′** molecular structures. (**b**) Molecular electrostatic potential (MEP) of Complex **1′** and Complex **2′,** calculated at the level of theory PBEPBE/LANL2DZ.

Figure 5a,b show the optimized structures and the molecular electrostatic potential (MEP) of complexes **1′** and **2′**, respectively, if the hydrolysis of the polymers is considered. In Figure 5a, in Complex **1′**, the Cu1 atom is coordinated in the equatorial sites with the citrulline molecule with distances of Cu1–O1 1.991 Å and Cu1–N1 2.049 Å, and with the 2,2-bipyridine molecule with distances of Cu1–N2 and Cu1–N3 2.008–2.040 Å. For Complex **2′**, the distances of Cu1–O1 1.992 Å and Cu1–N1 2.043 Å with citrulline are very similar and in the same form with the 1,10-phenanthroline molecule, with distances of Cu1–N2 and Cu1–N3 2.017–2.062 Å. These parameters at the equatorial positions coordinated with Cu(II) are in accordance with those previously calculated: for D, L-Citrullinato-bipyridine complex (Cu1–O1 2.021–2.024 Å, Cu1–N1 2.030–2.031 Å, Cu1–N2 2.007–2.012 Å, and Cu1–N3 2.034–2.038 Å) [24] for bis-Citrullinato complex (Cu1–O1 1.964–1.980 Å, Cu1–N1 2.018–2.027 Å) [38]; for L-Glutamine and phenanthroline complex (Cu1–N2 1.989–2.006 Å, and Cu1–N3 2.014–2.037 Å) [54]; and for Metformin and bipyridine complex (Cu1–N2 2.031 Å, and Cu1–N3 2.044 Å) [67]. On the other hand, in both complexes, the axial position of the distorted square pyramidal center is occupied by one H_2_O molecule, which is coordinated with Cu1 through O3, with Cu1–O3 distances of 2.278 and 2.280 Å for complexes **1′** and **2′**, respectively. Similar values of 3.019–3.030 Å for D, L-Citrullinato-bipyridine complex [24]; 2.221–2.308 Å for L-Glutamine and phenanthroline complex [54]; and 2.756 Å for Metformin and bipyridine complex [67], have been calculated for complexes with H_2_O molecule in the axial position coordinated with Cu(II). In Figure 5b, the molecular electrostatic potential (MEP) was mapped on the total electronic density in a range of −2.0 × 10^−2^ (red regions) to 2.0 × 10^−2^ (blue regions) of electronic density, with an isovalue = 0.0004 a.u. For complexes **1′** and **2′**, the negative charge density regions (nucleophilic zones) are located on the carboxylate and ureide groups of citrulline. In contrast, the deficient charge density regions (electrophilic zones) are located on the protons of the ureide, 2,2-bipyridine, and 1,10-phenanthroline. Intermediate electron density zones are shown in yellow and green regions. These electrophilic and nucleophilic regions are susceptible to interaction, including non-covalent interactions, with adjacent chains forming the supramolecular structure.

**Figure 6:**
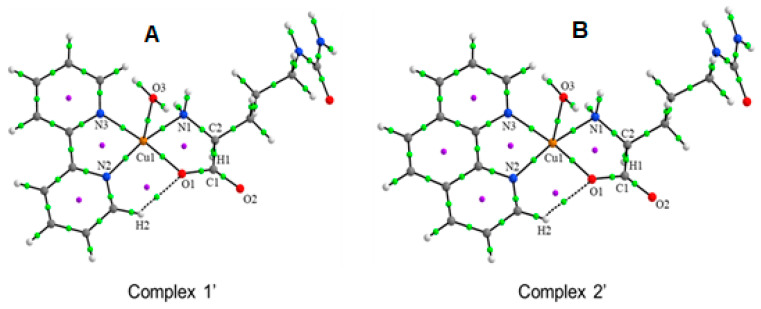
Molecular graphs of (**A**) Complex **1′** and (**B**) Complex **2′** showing the main BCPs and RCPs.

The non-covalent interactions were analyzed by topological parameters, such as the electron density, ρ(r), the Laplacian of density, ∇^2^ρ(r), and the energy of interaction, E_H···Y_. The results are shown in Table 4. Figure 5 shows Complex **1′** and **2′** molecular graphs, where the green dots indicate bond critical points (BCPs), and purple dots indicate ring critical points (RCPs). The values of ρ(r) on the BCPs are at the similar ranges 0.0747–0.0812 a.u. and 0.0747–0.0793 a.u., for Complex **1′** and **2′**, respectively, for the interactions in the equatorial positions coordinated with the Cu1 atom (Cu1···O1, Cu1···N1, Cu1···N2, and Cu1···N3), see Table 4. In addition, the positive values of ∇^2^ρ(r) in these interactions indicate the presence of metal-ligand non-covalent interactions in the equatorial positions coordinated with the Cu1 atom in both compounds. The calculated interaction energy (E_H···Y_) is in the range of 35.23–41.08 and 33.85–39.69 kcal mol^−1^ for Complex **1′** and Complex **2′**, respectively, with slightly higher values for Complex **1′** with 2,2-bipyridine, in the equatorial positions coordinated of the Cu1 atom. Similar values of interaction energies have been calculated for D, L-Citrullinato-bipyridine complex (33.95–40.73 kcal mol^−1^) [24], bis-Citrullinato complex (40.47–44.43 kcal mol^−1^) [38], L-Glutamine and phenanthroline complex (31.31–50.29 kcal mol^−1^) [54], Metformin and bipyridine complex (35.61–47.31 kcal mol^−1^), and Imidazol-pyridine and Glycine complex (37.87–42.20 kcal mol^−1^) [67], all of them in the equatorial sites coordinated with Cu(II). Concerning the axial position, the value of interaction energy for the interaction between Cu1 and the O3 of the H_2_O molecule, Cu1···O3, is 13.99 and 13.87 kcal mol^−1^ for Complex **1′** and **2′**, respectively. Similar values have been found for L-Glutamine and phenanthroline complex (13.02–16.44 kcal mol^−1^) [54], and for Imidazol-pyridine and Glycine complex (17.23 kcal mol^−1^) [67], for the interaction between a H_2_O molecule coordinated with Cu(II) in axial position. A weak interaction, H2···O1, is observed in both complexes with small value interaction energy (2.73 and 1.98 kcal mol^−1^). Several RCPs are observed (purple dots in Figure 6) forming stable ring structures of five atoms around Cu(II) coordinated in Complexes **1′** and **2′**.

### 3.3. Molecular Docking

**Figure 7:**
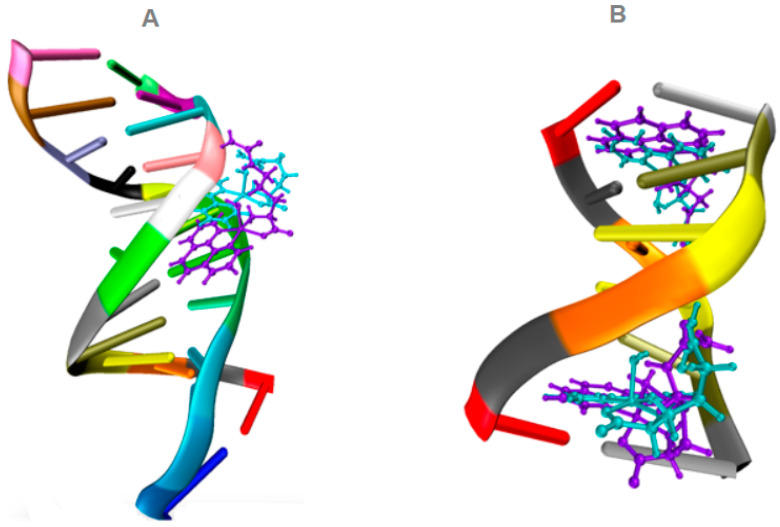
Docked structures of the top molecular poses of the two copper complexes under investigation: [Cu(L-Citr)(phen) (H_2_O)]^+^ in purple and [Cu(L-Citr)(bipy)(H_2_O)]^+^ in cyan, docked with 1BNA/DNA (**A**) and 151D/DNA (**B**).

Table 5 displays the docked binding energies corresponding to the copper complexes’ top molecular pose (lowest energy) presented for the docked complexes with DNA. As previously reported, these results can be compared with the Doxorubicin (DOX) reference ligand [54,55]. DOX is an anthracycline commonly employed as a chemotherapeutic drug. DOX exhibits a binding energy of −11.3 kcal/mol with DNA. When examining the interactions with DNA, the copper complexes exhibit lower binding energies than DOX. Nevertheless, both complexes interact well with the DNA molecule, occupying similar positions in the minor groove of the 1BNA/DNA and intercalating in the 151D/DNA fragment structures, as illustrated in Figure 7. Notably, Complex **2′** [Cu(L-Citr)(phen)(H_2_O)]^+^ exhibits the best binding energy among the two copper complexes. Interestingly, the copper complexes studied interact with the same nucleotides as DOX in 151D/DNA, although the nature of the interaction differs between them. This variation in interaction type could explain why these copper complexes demonstrated lower binding energies than those observed with DOX.

### 3.4. In-Vitro Experiments

#### 3.4.1. UV-Vis Experiments with CT-DNA

**Figure 8:**
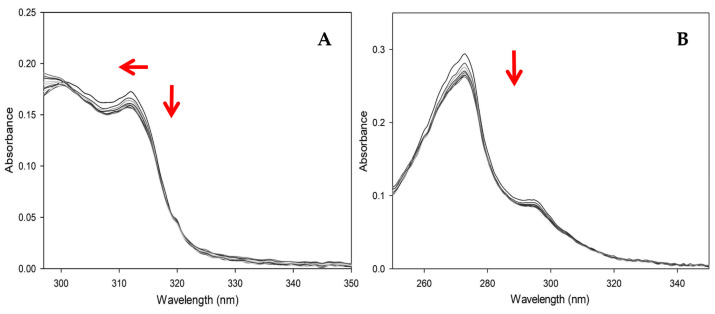
Electronic absorption spectra of Complex **1** (**A**) and Complex **2** (**B**) when titrated with CT-DNA. Every line represents a point in the titration, and the red arrows indicate the changes in the intensity and position of the complex bands in the experiment.

The electronic absorption spectra of complexes **1** and **2** (in water solution) are shown in Figure 8, in the range 250–350 nm, with each colored line representing a point on the titration curve. In Complex **1**, the addition of CT-DNA causes a decrease in intensity in the band at 310 nm (hypochromic effect) as the CT-DNA concentration increases and a slight blue shift (hypsochromic shift) by about 2 nm. However, in the band at 300 nm, although a decrease in intensity is experienced at the first points of the titration, the trend changes again until an increase in intensity is perceived. This results in a hyperchromic effect, followed by a shift and distortion of the band. The broad absorption band of DNA is separated at 40 nm from the band of Complex 1, and the above effect can be rationalized as a band overlap. 

In Complex **2**, due to the band overlap between the absorption band of DNA (260 nm) and the band of the complex (272 nm), the concentration of the CT-DNA solution had to be decreased one-fold. However, the first thing to observe is a gradual decrease in intensity over the band at 272 nm as the concentration of CT-DNA increases, resulting in a moderate hypochromic effect on the band. The band near 290 nm also experiences the same phenomenon but to a lesser extent. In both cases, there is no shift in the bands. According to several authors [59,64,68,69,70,71,72], the hypochromic effect on the visible spectrum, accompanied by a shift to both blue and red, indicates the binding of the molecule to DNA, implying that the complex tends to bind to DNA. However, the absence of a shift in Complex **2** leaves doubt about the interaction. Although it is possible to rationalize this absence as an effect of titrant dilution, this argument needs further support. 

**Figure 9:**
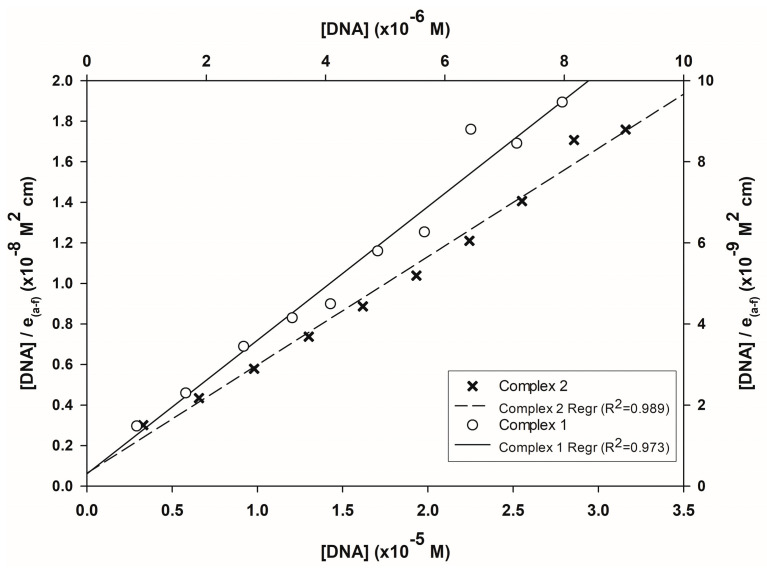
Wolfe-Schimer plot for the binding data of both complexes.

According to the linearized model of the Wolfe-Schimer equation (Figure 9), the value of the association constant (K_b_) for Complex **2** is 8.32 × 10^5^, and for Complex **1** is 3.76 × 10^4^, indicating a difference of one order of magnitude. Compared to other writers, the value is in the expected range for the mixed amino acid family (See Section 4), and the data from this experiment indicate that the complexes can bind DNA.

#### 3.4.2. Fluorometric Experiments with CT-DNA 

**Figure 10:**
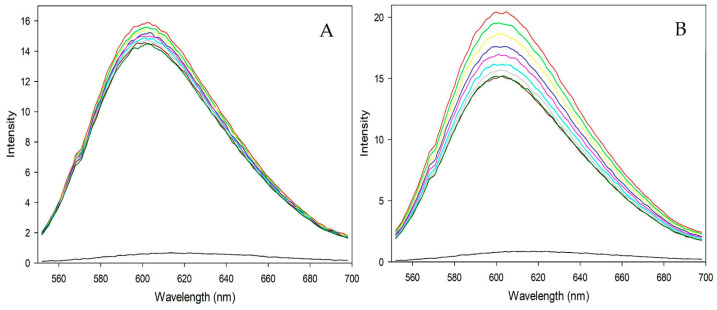
Fluorescence emission spectra of the EB-DNA complex titrated with Complex **1** (**A**) and Complex **2** (**B**).

Previously, the findings in the previous section showed a DNA binding affinity for the complexes, but how the two molecules bind cannot be easily explained. The fluorescent emission spectra from the titration experiment of the EB-DNA complex with metallic complexes **1** and **2** are shown in Figure 10. If the complexes can intercalate and displace EB, a decrease in intensity should be noted, which is observed in both cases. As the concentration of the complexes in the experiment increases, the measured intensity gradually decreases. This behavior in the experiments supports intercalation as a likely way for the synthesized complexes to bind to DNA. 

**Figure 11:**
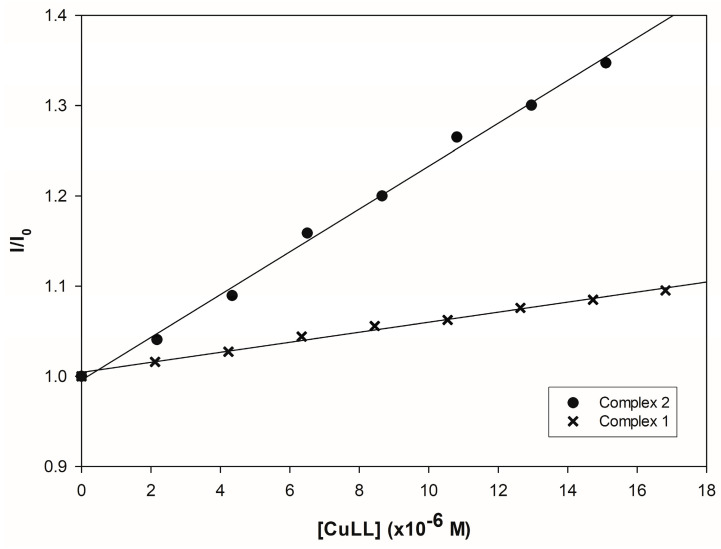
Stern-Volmer plots for the fluorescence data of both complexes.

Although both complexes show a reduction in intensity, Complex **2** shows more active behavior. Using the Stern-Volmer model on the fluorescence data, the K_sv_ constant acquires values of 5.57 × 10^3^ and 2.37 × 10^4^ for complexes **1** and **2,** respectively, representing a difference of one order of magnitude between both molecules (Figure 11). The variation in the values shown is mainly due to the type of tertiary ligand in the molecules. It should be noted that the theory states that the larger, bulkier, and more hydrophobic the functional group, the more favorable the insertion into the grooves of the double-stranded DNA molecule [64], and the analyzed complexes appear to be no exception.

#### 3.4.3. Interaction with pUC19

**Figure 12:**
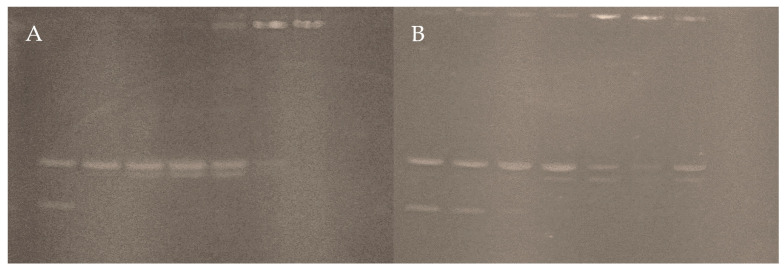
Electrophoresis of pUC19 plasmid were treated with Complex **2** (**A**) and Complex **1** (**B**), respectively. In both experiments, the complex concentration per lane equals 0, 5, 10, 15, 30, 50, and 100 μM, from left to right.

The electrophoresis bands of plasmid pUC19 treated with both complexes are shown in Figure 12. In both cases, the first lane corresponds to the untreated plasmid. The supercoiled form (Form II) is visible in the lower band, and the relaxed form (Form I) in the upper band, both without abnormalities. 

Linear DNA (form III) is seen in the experiment with Complex **2**. This occurs as a small splitting of the form II band from the lane at 5 μM, with a slight decrease in the intensity of the same band and the disappearance of the form I band. The above suggests that the complex can break the circular structure. Then, the same pattern is observed in the three lanes corresponding to concentrations between 10 and 30 μM, with a gradual increase in the intensity of Form III. However, when the concentration exceeds 50 μM, the intensity of forms II and III decreases entirely in the last lanes. Thus, it indicates that most of the DNA load cannot leave the well of the gel. From this concentration point, the DNA condenses due to a change in electrophoretic mobility caused by the cations of the complex neutralizing the negative charges of the molecule [73,74,75]. 

On the other hand, Complex **1** has similar behavior on the plasmid, but form III bands can be seen up to the lane corresponding to 15 μM. Although the form I is discernible in all bands, it is difficult to differentiate in the lowest concentration lane. Complex **2** produces the same condensed shape, unable to migrate on the gel at concentrations at or above 30 μM. Complex **2** loses the band corresponding to the circular relaxed form at 5 μM, whereas Complex **1** loses the band corresponding to the circular relaxed form at a threefold higher concentration. This again supports the power of a bulkier, hydrophobic ligand on DNA affinity.

#### 3.4.4. Cytotoxic Assays 

The IC_50_ values calculated for the two complexes compared to the four cell lines studied are summarized in Table 6. The inhibition values agree with the DNA affinity experiments: Complex **2** shows higher activity against cancer lines than Complex **1**, which shows inhibitory concentrations exceeding the 10 μM range. Activity against all four lines is favorable in Complex **2**, with HeLa cells being the most active (IC_50_ = 2.53 μM), followed by MCF-7, HCT-15, and MDA-MB-231 lines in ascending order. All the lines studied in the experiment correspond to different epithelial tissues, such as cervical (HeLa), breast (MDA and MCF-7), and colorectal (HCT-15), indicating that the complex has a broad spectrum of action. 

**Figure 13:**
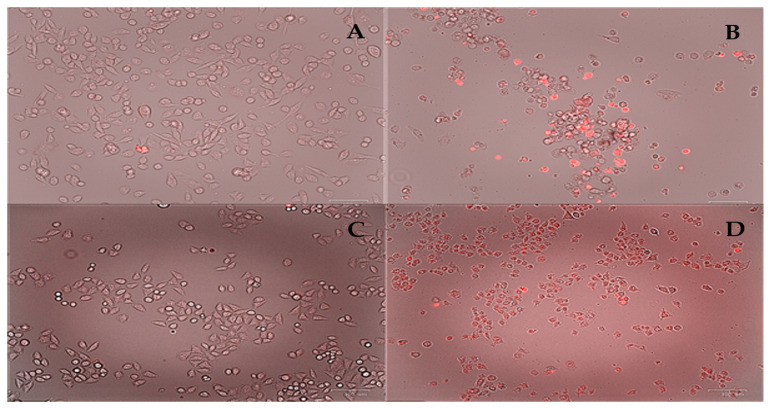
Fluorescent micrographs (10×) of HeLa and MDA-MB-231 cells treated with the Complex **2**. Micrographs (**A**,**C**) correspond to HeLa and MDA-MB-231 controls, respectively, while micrographs (**B**,**D**) correspond to HeLa and MDA-MB-231 treatments with the complex (10 μM) at 24 h. Propidium iodide staining was used.

On the other hand, Complex **1** has a relatively mild action. Viability experiments show a slight decrease in viability and mild activity against the HCT-15 line (IC_50_ = 11.77 μM). It can be concluded that the lines studied with this complex are very resistant to the molecule’s action. Complex **2** can alter the spindle-shaped morphology of HeLa and MDA cells, forming smaller circular clusters with high dye penetration, characteristic of apoptotic cell populations [76,77]. This can be seen with propidium iodide staining (Figure 13). This suggests that the complex’s ability to induce cell death is the cause of the loss of viability. 

## 4. Discussion

The main point to consider in the structures presented here is the formation of polymeric structures. In previous work, this was attributed to the ditopic nature of citrulline, which can bind a metal center with both the amino acid moiety and the ureido moiety [38]. In contrast to the [Cu(bipy)(Citr)(H_2_O)(NO_3_)](H_2_O) complex [24], the two complexes in this work maintain apical positions occupied by the ureido moiety. It should be recalled that in the synthesis of the previous work, the complex was recrystallized in water, and since this is the only difference in the synthesis of both complexes, it is most likely a ligand displacement phenomenon. A feasible explanation indicates water as a stronger sigma donor than the ureido group. 

Performing a search in the CCDC database (Figure S1 and Table 7) for aminoacidic Casiopeina analogs, there are more than 258 entries with phenanthroline and derivatives as the primary ligand, of which only ten correspond to polymeric complexes [78,79,80,81,82,83,84,85,86]. A common factor in all complexes, referred to as the main requirement for forming polymers, is the presence of more than one electron donor group, the carbonyl group (CO) being the most frequent. For this reason, ditopic ligands prefer to form polymeric structures. However, this is not a general rule since bridging ligands, such as nitrate ions (NO_3_^−^), can also form polymeric structures without coordinating the other moiety of the secondary ligand to another metal center [80,81,87]. A similar analysis of the 134 bipyridine analogs (Figure S2 and Table 8) shows similar patterns, but in these cases, the list is longer, with 11 molecules [87,88,89,90,91,92,93,94,95,96]. Similarly, most of the bonds are through an oxygen atom, present either in carbonyl groups or coming from the counterions of the molecule, and bonding is also observed through nitrogen atoms coming from both rings and amino groups. 

As mentioned, recrystallization in water is attributed as the main reason for forming a monomeric compound in the previous work. Therefore, when comparing the crystallization solvents of the examples shown in the tables, it can be observed that almost all of them are synthesized and crystallized in aqueous solutions of polar solvents. The proportion of water in the medium seems to affect the formation of polymeric structures significantly. Although, of course, the presence of water itself is not a mutually exclusive condition for forming polymeric structures since there are hydrated structures in the examples [78,80,81,83,84,90,91,92,94,95,96]. What seems to be an exclusive tendency when more than two bidentate ligands are involved is the coordination of the metal center by a water molecule. In octahedral geometries, chain formation can occur with one water molecule coordinated in an apical position, leaving the other position free for chain coordination, as observed in some instances. Nevertheless, if the geometry of the complex obeys a square-based pyramid arrangement, the fact that a water molecule occupies the apical position involves a blockage to chain formation. This is because water cannot coordinate to more than two metal centers. In terms of geometry, there does not seem to be a general pattern that covers all cases. In some cases, molecules with octahedral geometry and molecules with square pyramid geometry can be observed in the same crystal. However, an important observation is that octahedral geometries are observed when there are bridges with the counterion of the complex or the ligand has a denticity greater than 3 [80,81,82,86,87,90,93,94,95]. Square-based pyramid geometries are mainly observed when the denticity of the ligands is not greater than 3.

Regarding the “in vitro” highlights, there are some points to review. Spectrophotometric assays with CT-DNA show that both complexes have an affinity for the biomolecule, with an order of magnitude difference between bipyridine and phenanthroline, which is mainly attributed to the extra ring in the phenanthroline structure. The explanation of why this happens has been widely discussed by many authors, being a phenomenon attributable to the aromaticity and charge density of the ligand [59,64,69,97]. Fluorometric assays show evidence of the ability of both complexes to intercalate between DNA bases; similarly, experiments with plasmid DNA show that both complexes can cleave the structure. This information confers the term “metallonuclease” to the molecules in this study, although the exact mechanism by which this process occurs is not yet fully elucidated.

To describe the above point in more detail, it is necessary to provide some context. Copper (II) complexes have been known as potent nuclease-like compounds since the late 1970s, with the Sigman pioneering work about the activity of the ([Cu(phen)_2_]^2+^) complex on DNA [98,99]. This ability to cleave DNA strands occurs through the hydrolysis of the phosphodiester backbone or by extracting hydrogen atoms from the pentose ring, which is an oxygen-dependent process. The cleavage mechanism is related to the reactive oxygen species (ROS) through the oxidation of Cu(II) to Cu(III) in the presence of oxidizing agents such as hydrogen peroxide (H_2_O_2_) or molecular oxygen (O_2_). Likewise, the reduction of Cu(II) to Cu(I) using reducing agents such as ascorbic acid (C_6_H_8_O_6_) also produces ROS [100,101,102,103,104,105,106,107,108]. Most copper (II) complexes are known to cleave DNA through this mechanism [106], and within this group of compounds, the Casiopeinas are found. These compounds belong to a predominant family with several members known for their anticancer properties against various types of tumor cells both in laboratory settings (in vitro) and in living organisms (in vivo) [12,13,33,36]. Multiple hypotheses regarding the mechanism of action have been suggested, such as the excessive production of ROS through Fenton-type reactions, toxicity to mitochondria, and direct interaction with DNA [36,109,110,111]. Quantitative Structure-Activity Relationship (QSAR) investigations have demonstrated that the substituents of the ligands can alter the cytotoxicity and antiproliferative activity of Casiopeinas [36,109,110]. The complexes presented in this work show biological activity, and it is likely that the mechanism of action is closely related to the mentioned mechanisms. To support this idea, a comparison between the values obtained with other authors (Table 9) indicates that the affinity of the complexes is paired with values of Casiopeina with proven biological activity [69], while analogs similar to the citrulline structure, such as the complexes of Arginine, Ornithine, Asparagine, and Glutamine [79,111,112], show slightly lower values, but retain the same tendency of being the heterocyclic ligand with the highest charge density and the one with the highest biological activity. Again, a comparison with the complexes in Complex **9**, where assays similar to this work were carried out [79,112,113], also shows the same pattern, where the study complexes have the same ability to intercalate with DNA and cause cleavages to it. Although the values differ, the difference between heterocyclic ligands predominates over the difference between secondary ligands.

In cytotoxicity experiments, the values become more discrete, although, in this case, it is more difficult to compare results with those of other authors due to the cell lines and the controls used. The choice criteria vary between studies, but those using the same lines may be comparable. An example of the above is reported by Godínez-Loyola and Bravo-Gómez [110,114] with IC_50_ values in the HeLa line for [Cu(1,10-phen)(indo)](NO_3_) and [Cu(1,10-phen)(acac)](NO_3_) complexes of 2.30 and 10.7 μM respectively. Similarly, for the bipyridine analogs of these two complexes, [Cu(2,2′-bipy)(indo)](NO_3_) and [Cu(2,2′-bipy)(acac)](NO_3_), the authors report values of 25.2 and 42 μM respectively. Although the change of the indomethacin ligand over the acetylacetonate ligand reduces the inhibitory concentration by almost five times, the same pattern mentioned above is maintained.

The fact that the pattern is conserved in all the experiments carried out and is the same as that observed in various studies is not a surprise; there is a justification in the molecular structure of phenanthroline and bipyridine. It is interesting to highlight that, despite using a wide range of secondary ligands, the pattern does not change, indicating that the effects are additive concerning the secondary ligand. In the discussion of the structure of the complexes in this study, mention is made of the ability of water to block the formation of polymeric chains, which is evidence of the ability of this type of molecule. This lability may also indicate the lability of the secondary ligand bonds, as these bonds may break upon dissolution of the complexes in a physiological medium. This idea is not new in metal complexes; authors such as Allsopp and Ciancetta study ligand changes for carboplatin [115,116], while Costa-Pessoa and Correira do similar work in studying possible transformations in vanadium complexes [117,118]. Within the domain of copper compounds, the same approach was investigated. Ugone has suggested a predominant ligand replacement from reduced bioligands from cytosol and blood (e.g., glutathione (GSH), NADH, and ascorbate molecules) onto four copper (II) Casiopeina-type complexes [119]. Similarly, Costa-Pessoa suggests a ligand replacement and separation of phenanthroline from the copper atom [120]. Trends in “in vitro” assays, evidence about ligand lability in copper complexes, and background on ligand exchange in metal complexes suggest that Cassiopeia-type mixed copper complexes, in physiological media, should follow equivalent behavior.

This idea helps to explain the pattern mentioned above. Based on the fact that any casiopeina analog will decompose in physiological media by ligand substitutions, it is to be expected that the only ligand to remain attached to the metal center will be the heterocyclic ligand due to its strong field nature and reported stability constants [121]. Therefore, in any reaction with any analog, the copper-diimine moiety will be a common factor. Most of the observable biological activity is likely caused by the reaction product retaining the heterocyclic ligand. The secondary ligand, on the other hand, can be expected to be substituted relatively easily, considering that the above is not likely to be involved in a biologically active process, such as DNA damage. However, hydrogen bonds or hydrophobic interactions with side chains of the secondary ligands and DNA cannot be discarded. Although it is true that the biological activity values are different between analogs with different ligands, they all exert almost the same effects.

Several hypotheses can be formulated, but based on the data reviewed, it is possible to suggest that the function of the secondary ligand is to confer greater solubility to the final assembly. This hypothesis makes sense, considering the poor solubility of binary copper-diimine complexes, especially when bulky, hydrophobic substituents are present in the structure. Although it is less straightforward to speak of the solubility of the complex as a function of the secondary ligand, this idea reconciles the observed patterns. The secondary ligand may also exert an effect on activity, but under the perspective described, the effect would be expected to be additive to the effect of the heterocyclic product. However, this is an open question for further research in the near future.

## 5. Conclusions

Two catena complexes of copper based on bipyridine and phenanthroline with the amino acid citrulline were obtained and crystallized from a methanolic solution. Also, considering that the catena complexes suffer hydrolysis in an aqueous solution, the resulting aqua-complexes were studied with the DFT methodology (Complexes **1′** and **2′**). Docking studies using 1BNA and 151D small DNA fragments were performed. The interaction with CT-DNA was carried out by UV-Vis spectroscopic studies. The intercalation of the complexes was analyzed using Ethidium Bromide displacement. The nuclease activity was performed by electrophoresis using the plasmid pUC19. Cytotoxic activity was performed on HeLa, MDA-MB-231, HCT-15, and MCF-7 cancer cell lines.

Complex **1**, [Cu(L-Citr)(bipy)(NO_3_)]_n_, and Complex **2**, [Cu(L-Citr)(phen)(NO_3_)]_n_ are catena complexes due to the coordination of ureido groups. However, if recrystallized in water, monomeric aqua-complexes result. DFT calculations have indicated similar behavior of monomeric aqua-complexes with Bipyridine and Phenanthroline, even in different complexes containing bis-Citrullinato, L-Glutamine, Metformin, or Glycine, when they have been analyzed by their distribution of electronic density and non-covalent interactions coordinated with the Cu(II) in a square pyramidal arrangement. Thus, the monomeric complexes **1′** and **2′** were studied theoretically to understand their interaction with DNA through docking calculations. Both compounds have good affinity with the DNA test molecules, indicating that they can interact with the minor groove of DNA and intercalate with it. The phenanthroline derivative has better affinity due to hydrophobic, π-π interactions, and hydrogen bonds with the ureido group of citrulline. This suggests a better anticancer activity than the bipyridine compound. CT-DNA, pUC19, and cytotoxic studies confirmed these findings. Actually, the difference between the two complexes is one order of magnitude. The values of IC_50_ are in the range of many Casiopeinas and analogs.

Preclinical and clinical research have collected promising evidence to support the medicinal potential of copper complexes. The primary advantage of copper complexes is their ability to selectively reduce Cu(II) to Cu(I) compounds within cancerous cells that trigger cell death. The potential of copper complexes in therapeutic applications is significant due to their high efficacy and low systemic toxicity. While Cu complexes have shown significant promise as anticancer drugs, there is still a need for a more methodical and focused approach for a copper (Cu) complex to transition from academic research to the therapeutic setting. These complexes show interesting biochemical and molecular pathways, which have helped move several potential copper-based cancer treatments into early clinical trials.

## Figures and Tables

**Table 1:** Crystallographic data and structure refinement details.

	Complex 1	Complex 2
Empirical formula	C_16_H_20_CuN_6_O_6_	C_18_H_20_CuN_6_O_6_
CCDC	2341818	2341817
Formula weight (g/mol)	455.92	479.94
Temperature (K)	293(2)	293(2)
Crystal system	Monoclinic	Triclinic
Space group	*P*2_1_	*P*1
a (Å)	5.5321(2)	9.2213(3)
b (Å)	9.1050(3)	9.4194(3)
c (Å)	18.6006(6)	12.3091(4)
a (°)	90	93.660(3)
β (°)	92.816(3)	93.753(3)
γ (°)	90	110.190(3)
Volume (Å^3^)	935.78(6)	997.04(6)
Z	2	2
*ρ*_calc_ (g/cm^3^)	1.618	1.599
μ (mm^−1^)	1.216	1.146
Radiation	MoKα (λ = 0.71073)	MoKα (λ = 0.71073)
Index ranges	−8 ≤ h ≤ 8, −14 ≤ k ≤ 14, −29 ≤ l ≤ 30	−14 ≤ h ≤ 14, −14 ≤ k ≤ 14, −18 ≤ l ≤ 18
Largest diff. peak/hole (e Å^−3^)	0.34/−0.47	0.42/−0.43
R_int_	0.0496	0.0482
GoF on F^2^	1.044	1.004
Final R indexes [I > 2σ(I)]	R_1_ = 0.0366, wR_2_ = 0.0652	R_1_ = 0.0461, wR_2_ = 0.0807
Final R indexes [all data]	R_1_ = 0.0647, wR_2_ = 0.0756	R_1_ = 0.0834, wR_2_ = 0.0934

**Table 2:** Lengths (Å) and angles (°) for selected hydrogen bonds in Complex **1**.

D-H···A	D-H Distance	H···A Distance	D···A Distance	Angle
N24-H24B···O26	0.89	2.289	3.103(3)	151.9
N24 ^i^-H24A···O14	0.89	2.347	3.236(3)	177.6
N20 ^ii^-H20···O23	0.86	2.158	2.947(3)	152.3
N22 ^ii^-H22A···O23	0.83(3)	2.37(3)	3.113(3)	149.7(3)
N22 ^iii^-H22B···O23	0.88(4)	2.32(4)	3.161(3)	158.2(3)

Symmetry operations: ^i^ = 1 + x, y, z; ^ii^ = 2 − x, ½ + y, 2 − z; ^iii^ = 1 + x, 1 + y, z.

**Table 3:** Lengths (Å) and angles (°) for selected hydrogen bonds in Complex **2**.

D-H···A	D-H Distance	H···A Distance	D···A Distance	Angle
N3-H3A···O7	0.89	2.26	3.004(5)	141.5
N3-H3B···O2 ^i^	0.89	2.41	3.100(4)	134.7
N4-H4···O12 ^i^	0.86	2.22	3.021(5)	154.7
N5-H5A···O2 ^ii^	0.86	2.08	2.916(5)	164.3
N5-H5B···O12 ^i^	0.86	2.38	3.152(6)	149.5
N8-H8A···O5	0.89	2.09	2.963(4)	165.6
N8-H8B···O10	0.89	2.18	3.013(4)	155.5
N9-H9A···O9	0.86	2.12	2.957(5)	163.3
N10-H10A···O5 ^iii^	0.86	2.15	2.990(5)	164.4
N10-H10B···O9	0.86	2.42	3.174(6)	147.3

Symmetry operations: ^i^ = x, y, 1 + z; ^ii^ = −1 + x, y, 1 + z; ^iii^ = x, −1 + y, z.

**Table 4:** Topological parameters (in a.u.) and interaction energies E_.H. ···Y_ (in kcal mol^−1^) of Complex **1′** and Complex **2′**. Atom labels correspond to those shown in Figure 4 and Figure 5.

Complex 1′			
BCP	ρ (r)	∇^2^ρ (r)	E_H···Y_
Cu1···O1	0.0747	0.4718	38.00
Cu1···N1	0.0760	0.3964	35.23
Cu1···N2	0.0812	0.4601	41.04
Cu1···N3	0.0753	0.4257	36.68
Cu1···O3	0.0377	0.2307	13.99
H2···O1	0.0124	0.0598	2.73
**Complex 2′**			
BCP	ρ (r)	∇^2^ρ (r)	E_H···Y_
Cu1···O1	0.0747	0.4711	37.90
Cu1···N1	0.0771	0.3997	35.85
Cu1···N2	0.0793	0.4500	39.69
Cu1···N3	0.0713	0.4035	33.85
Cu1···O3	0.0375	0.2289	13.87
H2···O1	0.0097	0.0472	1.98

**Table 5:** Binding energies for the best molecular poses of the two complexes studied.

Compound	Binding Energy (Kcal/mol)	
	DNA/1BNA	DNA/151D
Complex **1′**	−9.3	−7.7
Complex **2′**	−9.8	−9.1

**Table 6:** IC_50_ values (μM) of the complexes against cell lines.

Complex	Cancer Cell Line			
	HeLa	MCF-7	MDA MB 231	HCT-15
Complex **1**	>10			11.77 ± 0.61
Complex **2**	2.53 ± 0.04	4.67 ± 0.59	6.69 ± 0.60	5.20 ± 0.09

**Table 7:** phenanthroline-Copper (II) analogs with polymeric structure reported in the CCDC database.

Complex	Secondary Ligand	Coordinating Moiety	Crystallization Solvent	Ref.
[Cu(L-Trp) (1,10-phen)](ClO_4_)(H_2_O)	L-Trp	Trp α-CO (μ-O)	Water/EtOH	[78]
[Cu(Asn) (1,10-nphen)](ClO_4_)	L-Asn	Asn δ-CO (μ-O)	Water/MeOH	[79]
[Cu(Orn)(1,10-phen)](NO_3_)2(H_2_O)	L-Orn	*CI* (NO_3_) (μ-O)	Water/EtOH	[80]
[Cu(D/L-dhpg)(1,10-phen) (NO_3_)]x(H_2_O)	D/L-dhpg	*CI* (NO_3_) (μ-O)	Water/EtOH (5:3)	[81]
[Cu2(H_2_ttha)(1,10-phen)_2_]	(H_2_ttha)^−4^	-NAc_2_ (μ_4_-N, O)	Water/EtOH (4:1)	[82]
[Cu(3-pysaa)(1,10-phen)]_2_(H_2_O)	3-pysaa	Pyr (μ-N)	Water/EtOH (2:3)	[83]
[Cu(SMe-L-Cys)(dppz)(H_2_O)](NO_3_)	SMe-L-Cys	Cys α-CO (μ-O)	Water/MeOH (1:2)	[84]
[Cu(L-Asp)(1,10-phen)](H_2_O)	L-Asp	Asp δ-CO (μ-O)	Water/EtOH (1:1)	[85]
[Cu(sptc)(1,10-phen)]	(sptc)^−2^	TC (μ_2_-N, O)	MeCN/MeOH (5:4)	[86]

***CI*** = counterion; **1–10-nphen** = nitro-1,10-phenanthroline; **D/L-dhpg** = D(−)/L(+)-4-hydroxyphenylglycine; **H_2_ttha** = 1,3,5-triazine-2,4,6-triamine hexaacetic acid; **3-pysaa** = N-3-pyridine sulfonyl amino acid; **Sme-L-Cys** = S-methyl-L-cysteine; **dppz**= dipyrido [3,2-a:2′,3′-c]phenazine; **sptc** = 1,1′-[sulfonylbis(4,1-phenylene)]bis(1 H-1,2,3-triazole-4-carboxylate); **TC** = triazole-4-carboxylate moiety.

**Table 8:** bipyridine-Copper (II) analogs with polymeric structure reported in the CCDC database.

Complex	Secondary Ligand	Coordinating Moiety	Crystallization Solvent	Ref.
[Cu(L-Glu)(2,2′-bipy)]	L-Glu	Glu ε-COO (μ-O)	Water/MeOH (3:1)	[88]
[Cu(L-Arg)(2,2′-bipy)(NO_3_)](NO_3_)	L-Arg	*CI* (NO_3_) (μ-O)	Water/MeOH (2:1)	[87]
[Cu(Hdta)(2,2′-bipy)]	Htda^−2^	TA (μ-N)	MeCN	[89]
[Cu_2_(L-Arg)_2_(2,2′-bipy)_2_(ClO_4_)_2_]2(ClO_4_)4(H_2_O)	L-Arg	*CI* (ClO_4_) (μ2-O)	Water/EtOH	[90]
[Cu_4_(L-Cys-Cys)(2,2′-bipy)_4_]4(ClO_4_)1.5(H_2_O)	L-Cys-Cys	Cys α-COO(μ-O)	MeOH	[91]
[Cu(L-NH_2_Phe)(2,2′bipy)](NO_3_)(H_2_O)	L-NH_2_-Phe	-NH_2_ (μ-N)	Water/MeOH	[92]
[Cu(H_2_adip)_2_(2,2′-Bipy)]	adip^−2^	MeCOO (μ-O)	Water	[93]
[Cu(D,L-Lys)(2,2′-bipy)]_2_(V_4_O_10_)7(H_2_O)	D, L-Lys	*CI* (V_4_O_10_) (μ-O)	Water	[94]
[Cu(L-Glu)(2,2′-bipy)(H_2_O)] [Cu(L-Glu)(2,2′-bipy)(ClO_4_)](ClO_4_)2(H_2_O)	L-Glu	*CI* (ClO_4_) (μ-O)	MeOH	[95]
[Cu(L-Gln)(2,2′-bipy)(H_2_O)] [Cu(L-Gln)(2,2′-bipy)](SO_4_)4(H_2_O)	L-Gln	L-Gln α-COO (μ-O)	Water/MeOH	[96]

***CI*** = counterion; **Htda** = 1,2,3-triazole-4,5-dicarboxylic acid; **TA** = Triazole moiety; **L-Cys-Cys** = L-Cystine; **L-NH_2_-Phe** = 4-Aminophenylalanine; **H_2_adip** = 5-Aminodiacetic isophthalic acid.

**Table 9:** Affinity Constants (K_b_) for several Casiopeina analogs.

Compound	K_b_ (M^−1^)	Reference
[Cu(L-Citr)(1,10-phen)(H_2_O)]NO_3_ (**Complex 2′**)	8.32 × 10^5^	This work
[Cu(L-Citr)(2,2′-bipy)(H_2_O]NO_3_ (**Complex 1′**)	3.76 × 10^4^	
[Cu(2,2′ -bipy)(gly)(H_2_O)]NO_3_ (**CasVII-gly**)	3.23 × 10^5^	[69]
[Cu(4,4-dimethyl-2,2′-bipy)(acac)(H_2_O)]NO_3_ (**CasIII-ia**)	3.31 × 10^5^	
[Cu(1,10-phen)(gly)(H_2_O)]NO_3_ (**CasVII-gly**)	7.15 × 10^5^	
[Cu(phen)(acac)(H_2_O)]NO_3_ (**CasIII-Ba**)	7.60 × 10^5^	
[Cu(4,7-dimethyl-1,10-phen)(acac)(H_2_O)]NO_3_ (**CasIII-Ea**)	7.90 × 10^5^	
[Cu(5,6-dimethyl-1,10-phen)(gly)(H_2_O)]NO_3_ (**CasVI-gly**)	7.89 × 10^5^	
[Cu(L-Arg)(2,2′-bipy)(Cl)](Cl)	1.70 × 10^3^	[112]
[Cu(L-Orn)(1,10-phen)(Cl)](Cl)	2.70 × 10^4^	[113]
[Cu(L-Asn)(1,10-nphen)](ClO_4_)	2.43 × 10^3^	[79]
[Cu(L-Gln)(1,10-nphen)(H_2_O)](ClO_4_)	1.05 × 10^4^	
[Cu(L-Gln)(1,10-phen)(H_2_O)](NO_3_)(H_2_O)	3.62 × 10^3^	[113]
[Cu(L-Gln)(dmphen)(H_2_O](ClO_4_)	7.33 × 10^3^	

## Data Availability

Data are contained within the article.

## Supplementary Materials

The following supporting information can be downloaded at https://www.mdpi.com/article/10.3390/pharmaceutics16060747/s1, CheckCIF proof of Complex **1**. CheckCIF proof of Complex **2**; Table S1: Media used for the maintenance of the cell lines in this study; Figure S1: ConQuest query to phenanthroline Casiopeina-type analogs; Figure S2: ConQuest query to bipyridine Casiopeina-type analogs.
