# Supplementary material for: L-Citrullinato-Bipyridine and L-Citrullinato-Phenanthroline Mixed Copper Complexes: Synthesis, Characterization and Potential Anticancer Activity
Source: Pharmaceutics. 2024 May 31;16(6):747. doi: 10.3390/pharmaceutics16060747 (PMC11207372; doi:10.3390/pharmaceutics16060747)
Supplement: Supplementary file 1 [file pharmaceutics-16-00747-s001.zip › pharmaceutics-3019514-supplementary.pdf]

Supplementary Materials

L-Citrullinato-Bipyridine and L-Citrullinato-Phenanthroline Mixed Copper Complexes: Synthesis, Characterization and Potential Anticancer Activity

Diego Ramírez-Contreras [1], Sergio Vázquez-Rodríguez [1], Amalia García-García [1,2], Lisset Noriega [3], Angel Mendoza [1], Brenda L. Sánchez-Gaytán [1], Francisco J. Meléndez [4], María Eugenia Castro [1], Maura Cárdenas-García [5,*] and Enrique González-Vergara [1,*]

CheckCIF report for **Complex 1** $[Cu(L\text{-}Citr)(bipy)(NO_3)]_n$

checkCIF/PLATON report

Structure factors have been supplied for datablock(s) a61322_mo

THIS REPORT IS FOR GUIDANCE ONLY. IF USED AS PART OF A REVIEW PROCEDURE FOR PUBLICATION, IT SHOULD NOT REPLACE THE EXPERTISE OF AN EXPERIENCED CRYSTALLOGRAPHIC REFEREE.

No syntax errors found. CIF dictionary Interpreting this report

Datablock: a61322_mo

Bond precision:	C-C = 0.0042 A		Wavelength=0.71073
Cell:	a=5.5321(2)	b=9.1050(3)	c=18.6006(6)
	alpha=90	beta=92.816(3)	gamma=90
Temperature:	293 K		

	Calculated	Reported
Volume	935.78(5)	935.78(6)
Space group	P 21	P 1 21 1
Hall group	P 2yb	P 2yb
Moiety formula	C16 H20 Cu N6 O6	C16 H20 Cu N5 O3, N O3
Sum formula	C16 H20 Cu N6 O6	C16 H20 Cu N6 O6
Mr	455.93	455.92
Dx,g cm-3	1.618	1.618
Z	2	2
Mu (mm-1)	1.216	1.216
F000	470.0	470.0
F000’	470.83	
h,k,lmax	9,14,30	8,14,30
Nref	8423[4423]	7951
Tmin,Tmax	0.716,0.922	0.816,0.972
Tmin’	0.552	

Correction method= # Reported T Limits: Tmin=0.816 Tmax=0.972
AbsCorr = ANALYTICAL

Data completeness= 1.80/0.94 Theta(max)= 35.330

R(reflections)= 0.0366(5942) wR2(reflections)= 0.0756(7951)

S = 1.045 Npar= 271

The following ALERTS were generated. Each ALERT has the format
test-name_ALERT_alert-type_alert-level.
Click on the hyperlinks for more details of the test.

Alert level C

```
PLAT042_ALERT_1_C Calc. and Reported MoietyFormula Strings  Differ     Please Check
PLAT241_ALERT_2_C High   'MainMol' Ueq as Compared to Neighbors of        O29 Check
PLAT242_ALERT_2_C Low    'MainMol' Ueq as Compared to Neighbors of        N27 Check
```

Alert level G

```
PLAT004_ALERT_5_G Polymeric Structure Found with Maximum Dimension          1 Info
PLAT007_ALERT_5_G Number of Unrefined Donor-H Atoms .............           3 Report
PLAT199_ALERT_1_G Reported _cell_measurement_temperature ..... (K)        293 Check
PLAT200_ALERT_1_G Reported   _diffrn_ambient_temperature ..... (K)        293 Check
PLAT232_ALERT_2_G Hirshfeld Test Diff (M-X)  Cu1      --O25_b    .       12.5 s.u.
PLAT794_ALERT_5_G Tentative Bond Valency for Cu1        (II)      .      2.17 Info
PLAT883_ALERT_1_G No Info/Value for _atom_sites_solution_primary .     Please Do !
PLAT910_ALERT_3_G Missing # of FCF Reflection(s) Below Theta(Min).          3 Note
PLAT912_ALERT_4_G Missing # of FCF Reflections Above STh/L=  0.600        149 Note
PLAT978_ALERT_2_G Number C-C Bonds with Positive Residual Density.          4 Info
```

0 **ALERT level A** = Most likely a serious problem - resolve or explain
0 **ALERT level B** = A potentially serious problem, consider carefully
3 **ALERT level C** = Check. Ensure it is not caused by an omission or oversight
10 **ALERT level G** = General information/check it is not something unexpected

4 ALERT type 1 CIF construction/syntax error, inconsistent or missing data
4 ALERT type 2 Indicator that the structure model may be wrong or deficient
1 ALERT type 3 Indicator that the structure quality may be low
1 ALERT type 4 Improvement, methodology, query or suggestion
3 ALERT type 5 Informative message, check

It is advisable to attempt to resolve as many as possible of the alerts in all categories. Often the minor alerts point to easily fixed oversights, errors and omissions in your CIF or refinement strategy, so attention to these fine details can be worthwhile. In order to resolve some of the more serious problems it may be necessary to carry out additional measurements or structure refinements. However, the purpose of your study may justify the reported deviations and the more serious of these should normally be commented upon in the discussion or experimental section of a paper or in the "special_details" fields of the CIF. checkCIF was carefully designed to identify outliers and unusual parameters, but every test has its limitations and alerts that are not important in a particular case may appear. Conversely, the absence of alerts does not guarantee there are no aspects of the results needing attention. It is up to the individual to critically assess their own results and, if necessary, seek expert advice.

Publication of your CIF in IUCr journals

A basic structural check has been run on your CIF. These basic checks will be run on all CIFs submitted for publication in IUCr journals (*Acta Crystallographica*, *Journal of Applied Crystallography*, *Journal of Synchrotron Radiation*); however, if you intend to submit to *Acta Crystallographica Section C* or *E* or *IUCrData*, you should make sure that full publication checks are run on the final version of your CIF prior to submission.

Publication of your CIF in other journals

Please refer to the *Notes for Authors* of the relevant journal for any special instructions relating to CIF submission.

PLATON version of 06/07/2023; check.def file version of 30/06/2023

Datablock a61322_mo - ellipsoid plot

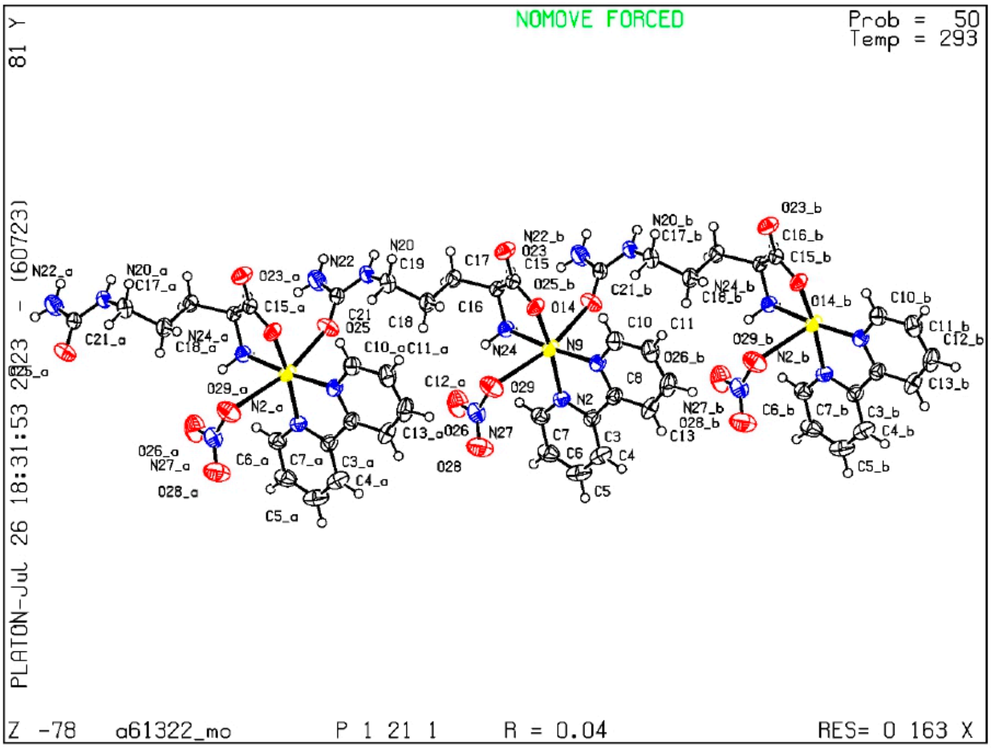

CheckCIF report for **Complex 2** [Cu(L-Citr)(phen)(NO_3)]$_n$

checkCIF/PLATON report

Structure factors have been supplied for datablock(s) a51322_mo

THIS REPORT IS FOR GUIDANCE ONLY. IF USED AS PART OF A REVIEW PROCEDURE FOR PUBLICATION, IT SHOULD NOT REPLACE THE EXPERTISE OF AN EXPERIENCED CRYSTALLOGRAPHIC REFEREE.

No syntax errors found.   CIF dictionary   Interpreting this report

Datablock: a51322_mo

```
Bond precision:   C-C = 0.0059 A           Wavelength=0.71073

Cell:            a=9.2213(3)        b=9.4194(3)       c=12.3091(4)
                 alpha=93.660(3)    beta=93.753(3)    gamma=110.190(3)
Temperature:     293 K

                  Calculated                   Reported
Volume            997.04(6)                    997.04(6)
Space group       P 1                          P 1
Hall group        P 1                          P 1
Moiety formula    C18 H20 Cu N6 O6             C18 H20 Cu N6 O6
Sum formula       C18 H20 Cu N6 O6             C18 H20 Cu N6 O6
Mr                479.95                       479.94
Dx,g cm-3         1.599                        1.599
Z                 2                            2
Mu (mm-1)         1.146                        1.146
F000              494.0                        494.0
F000'             494.84
h,k,lmax          14,14,18                     14,14,18
Nref              15222[ 7611]                 15179
```

```
Tmin,Tmax        0.708,0.751                  0.807,1.000
Tmin'            0.694

Correction method= # Reported T Limits: Tmin=0.807 Tmax=1.000
AbsCorr = MULTI-SCAN

Data completeness= 1.99/1.00        Theta(max)= 33.141

R(reflections)= 0.0461( 10511)              wR2(reflections)=
                                            0.0934( 15179)
S = 1.004                 Npar= 559
```

```
The following ALERTS were generated. Each ALERT has the format
       test-name_ALERT_alert-type_alert-level.
Click on the hyperlinks for more details of the test.
```

Alert level C

```
PLAT220_ALERT_2_C NonSolvent   Resd 1  O   Ueq(max)/Ueq(min) Range        3.8 Ratio
PLAT220_ALERT_2_C NonSolvent   Resd 2  O   Ueq(max)/Ueq(min) Range        3.3 Ratio
PLAT241_ALERT_2_C High   'MainMol' Ueq as Compared to Neighbors of        O12 Check
PLAT241_ALERT_2_C High   'MainMol' Ueq as Compared to Neighbors of        C35 Check
PLAT241_ALERT_2_C High   'MainMol' Ueq as Compared to Neighbors of         O9 Check
PLAT242_ALERT_2_C Low    'MainMol' Ueq as Compared to Neighbors of        N12 Check
PLAT242_ALERT_2_C Low    'MainMol' Ueq as Compared to Neighbors of        N11 Check
```

Alert level G

```
PLAT004_ALERT_5_G Polymeric Structure Found with Maximum Dimension          1 Info
PLAT007_ALERT_5_G Number of Unrefined Donor-H Atoms .............          10 Report
              H8A   H8B   H9A   H10A  H10B  H3A   H3B   H4    H5A   H5B
PLAT154_ALERT_1_G The s.u.'s on the Cell Angles are Equal ..(Note)      0.003 Degree
PLAT199_ALERT_1_G Reported _cell_measurement_temperature ..... (K)        293 Check
PLAT200_ALERT_1_G Reported   _diffrn_ambient_temperature ..... (K)        293 Check
PLAT794_ALERT_5_G Tentative Bond Valency for Cu1       (II)      .       2.11 Info
PLAT794_ALERT_5_G Tentative Bond Valency for Cu2       (II)      .       2.14 Info
PLAT933_ALERT_2_G Number of HKL-OMIT Records in Embedded .res File          4 Note
               -1 -1 -1,   0 -1  0,   1 -1  0,   4 10-10,
PLAT969_ALERT_5_G The 'Henn et al.' R-Factor-gap value ..........        1.71 Note
              Predicted wR2: Based on SigI**2  5.46 or SHELX Weight  9.47
PLAT978_ALERT_2_G Number C-C Bonds with Positive Residual Density.          2 Info
```

```
   0 ALERT level A = Most likely a serious problem - resolve or explain
   0 ALERT level B = A potentially serious problem, consider carefully
   7 ALERT level C = Check. Ensure it is not caused by an omission or oversight
  10 ALERT level G = General information/check it is not something unexpected

   3 ALERT type 1 CIF construction/syntax error, inconsistent or missing data
   9 ALERT type 2 Indicator that the structure model may be wrong or deficient
   0 ALERT type 3 Indicator that the structure quality may be low
   0 ALERT type 4 Improvement, methodology, query or suggestion
   5 ALERT type 5 Informative message, check
```

Validation response form

Please find below a validation response form (VRF) that can be filled in and pasted into your CIF.

```
# start Validation Reply Form
_vrf_PLAT220_a51322_mo
;
PROBLEM: NonSolvent   Resd 1  O   Ueq(max)/Ueq(min) Range        3.8 Ratio
RESPONSE: ...
;
_vrf_PLAT241_a51322_mo
;
```

```
PROBLEM: High   'MainMol' Ueq as Compared to Neighbors of        O12 Check
RESPONSE: ...
;
_vrf_PLAT242_a51322_mo
;
PROBLEM: Low    'MainMol' Ueq as Compared to Neighbors of        N12 Check
RESPONSE: ...
;
# end Validation Reply Form
```

It is advisable to attempt to resolve as many as possible of the alerts in all categories. Often the minor alerts point to easily fixed oversights, errors and omissions in your CIF or refinement strategy, so attention to these fine details can be worthwhile. In order to resolve some of the more serious problems it may be necessary to carry out additional measurements or structure refinements. However, the purpose of your study may justify the reported deviations and the more serious of these should normally be commented upon in the discussion or experimental section of a paper or in the "special_details" fields of the CIF. checkCIF was carefully designed to identify outliers and unusual parameters, but every test has its limitations and alerts that are not important in a particular case may appear. Conversely, the absence of alerts does not guarantee there are no aspects of the results needing attention. It is up to the individual to critically assess their own results and, if necessary, seek expert advice.

Publication of your CIF in IUCr journals

A basic structural check has been run on your CIF. These basic checks will be run on all CIFs submitted for publication in IUCr journals (*Acta Crystallographica*, *Journal of Applied Crystallography*, *Journal of Synchrotron Radiation*); however, if you intend to submit to *Acta Crystallographica Section C* or *E* or *IUCrData*, you should make sure that full publication checks are run on the final version of your CIF prior to submission.

Publication of your CIF in other journals

Please refer to the *Notes for Authors* of the relevant journal for any special instructions relating to CIF submission.

PLATON version of 06/01/2024; check.def file version of 05/01/2024

Datablock a51322_mo - ellipsoid plot

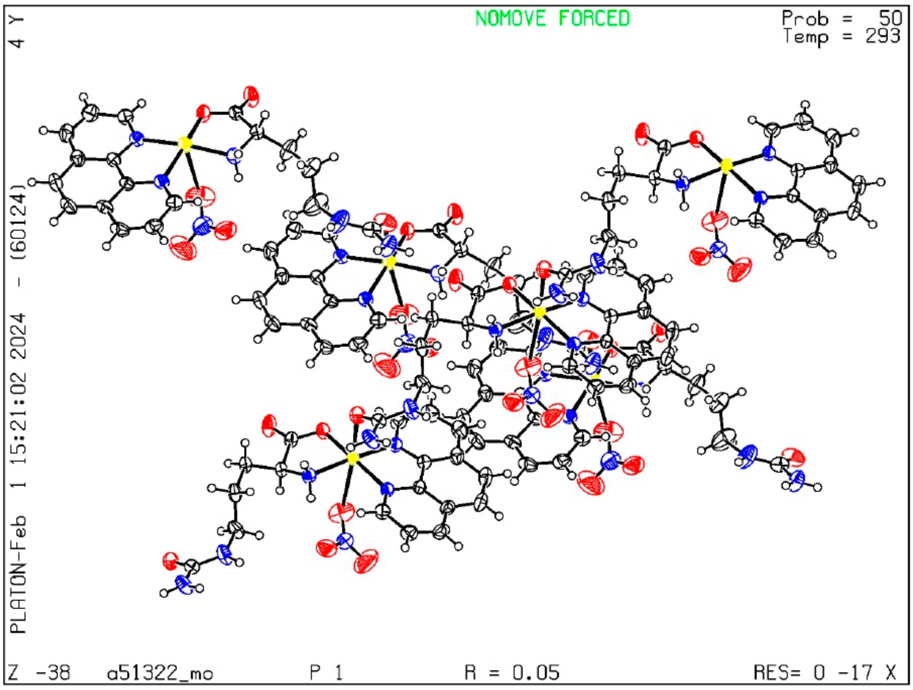

Table S1. Media used for the maintenance of the cell lines in this study

Cell Line	Cell culture medium	Suplemments
HeLa	Eagle´s Minimum Essential Medium (EMEM)	L-glutamine, Sodium Pyruvate, Glucose, without Phenol Red indicator, $NaHCO_3$ buffered, without HEPES buffer
MCF-7	DMEM/Ham´s F12 (1:1) Medium mixture	
MDA-MB-231	Dulbecco´s Modified Eagle Medium (DMEM)	
HCT-15	RMPI 1640 Medium	

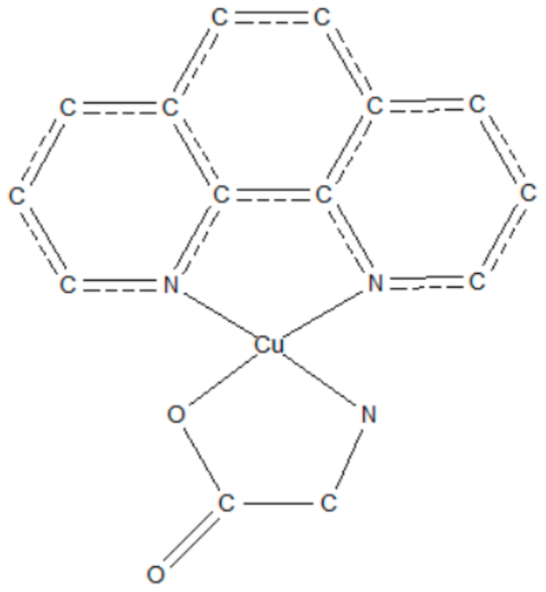

Figure S1. ConQuest query to phenantrholine Casiopeina-type analogs

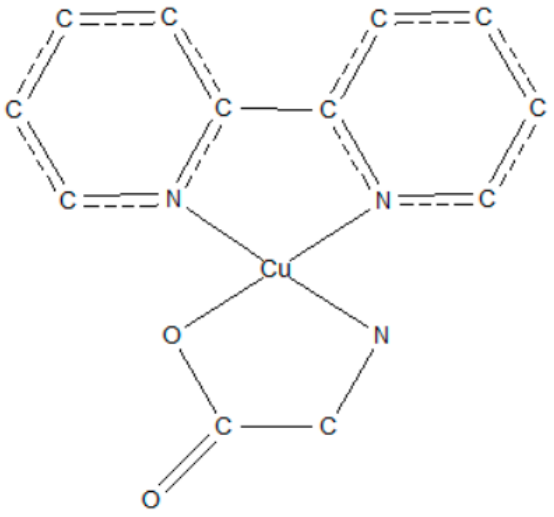

Figure S2. ConQuest query to bipyridine Casiopeina-type analogs
